# *Chlamydia* infection status, genotype, and age-related macular degeneration

**Published:** 2012-01-10

**Authors:** Sam Khandhadia, Sebastian Foster, Angela Cree, Helen Griffiths, Clive Osmond, Srinivas Goverdhan, Andrew Lotery

**Affiliations:** 1 Division of Clinical Neurosciences, University of Southampton, Southampton General Hospital, Southampton, UK; 2 Queen Alexandra Hospital, Cosham, UK; 3 MRC Epidemiology Resource Centre, University of Southampton, Southampton General Hospital, Southampton, UK; 4 Southampton Eye Unit, Southampton General Hospital, Southampton, UK

## Abstract

**Purpose:**

To evaluate whether *Chlamydia* (*C*.) infections are associated with age-related macular degeneration (AMD) and to assess if this association is influenced by the complement factor H (*CFH*) Y402H or the high temperature requirement A serine peptidase 1 (*HTRA1*) rs11200638 risk genotypes.

**Methods:**

One hundred ninety-nine AMD patients with early and late forms of the disease and 100 unaffected controls, at least 50 years old were included in the study. Patients in the AMD and control groups were selected based on known *CFH* Y402H variant genotype status (one third homozygous CC, one third heterozygous CT, and one third wild-type TT). Plasma from all patients and controls was tested for *C. pneumoniae*, *C. trachomatis*, and *C. psittaci* IgG seropositivity using a micro-immunofluorescent assay to establish previous infection status. Assays were conducted blind to risk genotypes and the results analyzed using univariate and multivariate (logistic regression) analysis.

**Results:**

IgG seropositivity to *C. pneumoniae* was most prevalent (69.2%, n=207), followed by *C. trachomatis* (7.4%, n=22) and *C. psittaci* (3.3%, n=10). No association was found between each of the three *Chlamydia* species IgG seropositivity and AMD status or severity (early/late). There was also no significant association between *Chlamydia* species IgG seropositivity and AMD status or severity, in patients carrying at least one *CFH* Y402H risk allele (C) or *HTRA1* rs11200638 risk allele (A), with univariate or logistic regression analysis.

**Conclusions:**

*Chlamydia* infection status does not appear to be associated with AMD status or severity. The presence of *CFH* Y402H and *HTRA1* rs11200638 risk genotypes does not alter this negative association.

## Introduction

Age-related macular degeneration (AMD) is the leading cause of severe visual impairment in developed countries [1,2], affecting approximately 30–50 million people worldwide (World Health Organization, Visual impairment and blindness). Environmental and genetic factors play a role in AMD pathogenesis [3-8]. However, the exact biochemical and cellular processes involved are not fully known. Several reports have described significant associations between complement genes and susceptibility to AMD. The genes include complement factor H (*CFH*) [9-12], complement 3 (*C3*) [13], high temperature requirement A serine peptidase 1 (*HTRA1*) [14], complement factor B (*CFB*) [15], and complement factor H-related 1+3 (*CFHR1*+*CFHR3*) [16,17]. A previous meta-analysis showed a 35% prevalence of the *CFH* Y402H (rs1061170, T→C) risk allele (C) in AMD. This increased the risk of AMD significantly (odds ratios of 2.5 and 6.3 for the heterozygous CT and homozygous CC genotypes, respectively) with an estimated population risk of 59% [18].

The association of *CFH* Y402H with AMD is intriguing as the CFH protein is involved in regulating the alternative complement pathway. By binding to C3b, the CFH protein accelerates the decay of the alternative pathway convertase C3bBb, and acts as a cofactor for complement factor I, another C3b inhibitor [19,20]. Activation of the alternative complement pathway is normally triggered by microbes, including the *Chlamydia* species [21-23]. This suggests that chronic low-grade *Chlamydia* infection in the presence of abnormal CFH protein production may lead to enhanced alternative complement pathway activation in the retina, therefore increasing an individual’s risk of developing AMD.

The *Chlamydiae* include three species that can infect humans: *Chlamydia (C.) pneumoniae*, *C. trachomatis*, and *C. psittaci*. *Chlamydiae* are obligate intracellular parasites, due to their reliance on host metabolism. They are found in the environment as non-active stable small cells known as elementary bodies (EB). These cells are able to bind to and enter host epithelial cells, forming larger intracellular reticulate bodies (RB). The RB then multiply, deriving energy from host metabolic processes, to form a cytoplasmic inclusion. This inclusion can then release new EBs from the host cell to infect other cells. Typically, *Chlamydiae* remain in the host on a subclinical level on a prolonged basis [24].

*C. pneumoniae* causes respiratory tract infections in humans, including pneumonia, bronchitis, pharyngitis, and sinusitis. *C. pneumoniae* is transmitted airborne, human to human. It is extremely prevalent, with 30%–50% of the population carrying *C. pneumoniae* antibodies worldwide. Only one species of *C. pneumoniae* has been described. Chronic infection with *C. pneumoniae* has been associated with AMD and other degenerative diseases (atherosclerosis [25-29], cardiac valvular stenosis [30], Alzheimer disease [31], and multiple sclerosis [32]). The association between *C. pneumoniae* and AMD is not fully established in the literature. Various studies, including preclinical and clinical studies, have all shown contradictory results (see the summary in Appendix 1) [33-44]. In addition, the association of C. *pneumoniae* with *CFH* polymorphisms in AMD has not been consistently replicated [40,42,45].

*C. trachomatis* can cause a range of diseases in humans, including trachoma, inclusion conjunctivitis, non-gonococcal urethritis, salpingitis, cervicitis, and lymphogranuloma venereum. *C. trachomatis* is transmitted person to person, including by sexual contact and from mother to baby during delivery. At least 15 antigen-specific species (“serovars”) of *C. trachomatis* have been described, including B, Ba, C-K, and L1-L3 [24]. The prevalence of *C. trachomatis* in a general European population aged 15–40 is around 3% [46], but can be up to 17% in young women [47]. *C. trachomatis* is endemic in poorer countries, where it is a leading cause of blindness through trachoma. Only one study has investigated the association between *C. trachomatis* and AMD but found no association [33]. No study has examined the association with the *CFH* genotype and *C. trachomatis* in AMD.

The natural hosts for *C. psittaci* are birds, especially parrots and parakeets. *C. psittaci* can be transmitted via bird excretions to humans, causing a disease known as psittacosis, which primarily causes atypical pneumonia. At least four serovars of *C. psittaci* have been described [24]. *C. psittaci* prevalence in the general population is the least common of the *Chlamydia* species, but is typically more common in bird handlers [48]. No studies have as yet investigated the association between *C. psittaci* and AMD.

In this study, we investigated the association of all three *Chlamydia* species (C. *pneumonia*, C. *trachomatis*, and *C. psittaci*) with the *CFH* Y402H AMD risk variant in AMD. We also investigated the association of the *Chlamydia* species with another genetic variant strongly associated with AMD, the *HTRA1* gene (rs11200638, G→A) [49]. The function of this gene is not yet known, and has not been previously associated with *Chlamydia*.

## Methods

Patients were selected from a preexisting database of AMD and control patients. Selection was restricted to those of Caucasian origin, at least 50 years old, and with confirmed genotyping [50]. Recorded height and weight were used to calculate body mass index (BMI). All patients were recruited under UK National Research Ethics Service approval, and had previously given full informed consent.

AMD status was determined according to the Age-Related Eye Disease Study (AREDS) grading system using clinical examination, stereoscopic fundus photographs, and fluorescein angiography (Topcon TRC50IX) [51]. AREDS grades 1–4 corresponded to AMD. AREDS grades 1–3 corresponded to early AMD, and AREDS 4 to late/advanced AMD.

Patients were selected based on AMD and *CFH* Y402H genotype status. In the AMD and control groups, one third of patients carried the homozygous (CC) risk *CFH* Y402H genotype, one third the heterozygous (CT) genotype, and the remaining one third the wild-type (TT) genotype. This was done to facilitate comparisons between genotypes. DNA was previously extracted using the salting-out method [52], and stored at −20 °C. *CFH Y402H* [50] and *HTRA1* rs11200638 genotype status were determined using TaqMan allelic discrimination assay probe kits on an Applied Biosystems 3730 Genetic Analyzer (Life Technologies Corporation, Carlsbad, CA).

### Determining plasma *Chlamydia* serology

Stored plasma samples were used for *Chlamydia* serology testing. These samples were previously obtained by collecting 10 ml of peripheral blood in lithium heparin tubes. These were then centrifuged within a few hours at 1,300 xg for 10 min, and the supernatant stored at −80 °C until analysis. Defrosted plasma samples were tested for the presence of *Chlamydia* IgG antibodies toward *C. pneumoniae*, *C. trachomatis*, and *C. psittaci* using a micro-immunofluorescent (MIF) assay kit (Focus Diagnostics, Cypress, CA). MIF is considered the laboratory gold standard for serological testing [53]. The IgG assay was selected as it indicates any previous *Chlamydia* infection and although titers do decrease after infection, the rate of decline is minimal.

**Figure 1:**
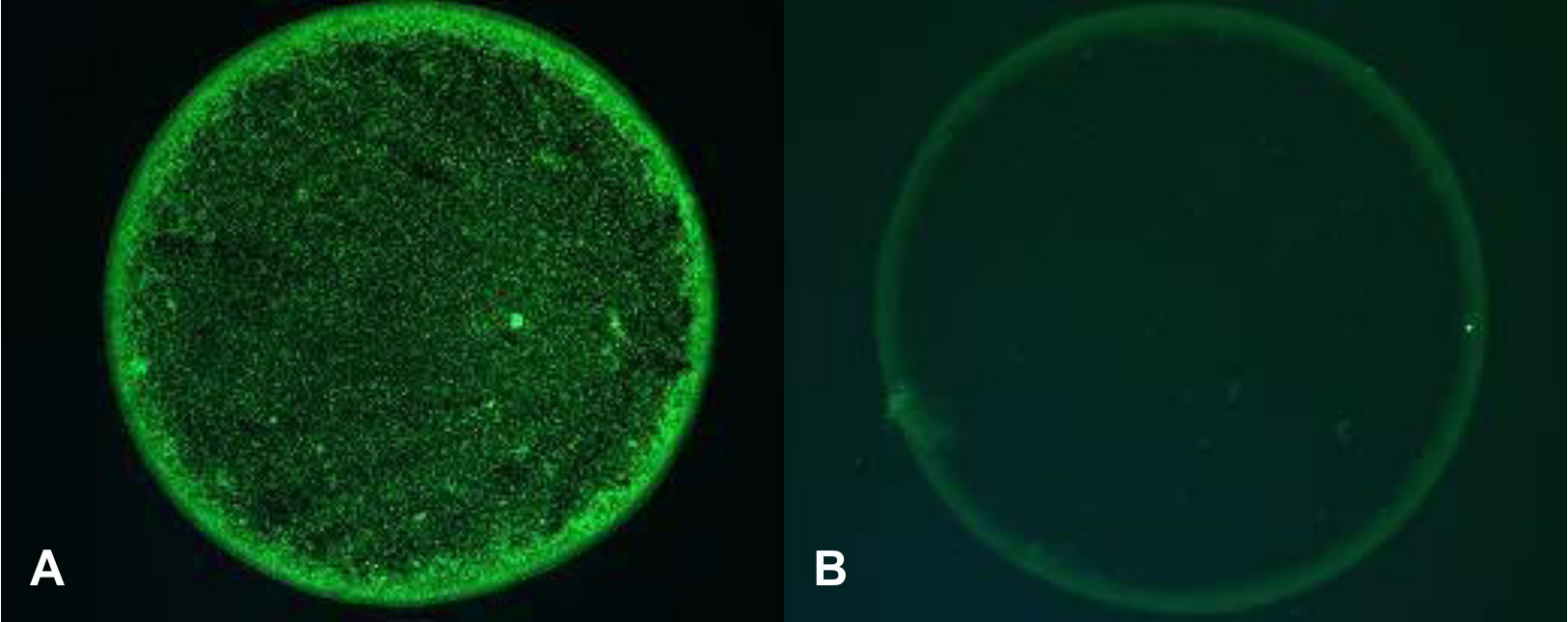
Positive and negative results from the MIF assay. The figure shows a spot within a well with positive (**A**) and negative (**B**) *C. pneumoniae* IgG seroreactivity as tested in this study using the Focus Diagnostics MIF assay (100× magnification).

The MIF assay was a two-stage “sandwich” procedure, using slides enclosed with the kit containing pretreated wells. A single well contained four individual spots. Three of these spots contained EBs of each *Chlamydia* species (each incorporating one strain of *C. pneumoniae*, two strains of *C. psittaci*, and eight serotypes (D-K) of *C. trachomatis*), diluted in 3% yolk sac (to provide background contrast). The fourth spot was composed of yolk sac alone and acted as a control. Each plasma sample was diluted 1:16 with phosphate buffered saline (PBS 0.01 M, containing sodium chloride 137 mM, phosphate buffer 10 mM, potassium chloride 2.7 mM), and 25 µl added to each well. The slide was covered and incubated at room temperature for 60 min. The slide was then washed with PBS for 10 min to remove unbound plasma antibodies, dipped in distilled water, and then air-dried. Twenty-five µl of fluorescein-labeled anti-IgG antibody was then added to each well. The slide was covered and re-incubated at room temperature for 30 min. The slide was then washed twice (as before). “Mounting medium” (supplied with the kit) was then added to the slide, and wells viewed using a fluorescence microscope at 400× magnification. Positive reactions appeared green, indicating fluorescent EBs on a background of yolk sac (Figure 1). A positive result was confirmed when the observed fluorescence level in any of the EB-containing spots was greater than that seen in the corresponding control yolk sac, no matter how minimal this difference was. A negative result was confirmed when there was no fluorescence, or the fluorescence equalled that observed in the corresponding yolk sac control spot or in the negative control well. Any samples with positive tests for all three *Chlamydiae* species were retested for verification.

The sensitivity and specificity of this test for detecting *C. pneumoniae* IgG seropositivity were specified as 62.5% and 99.8%, respectively; the corresponding values for *C. trachomatis* and *C. psittaci* were not available (Focus Diagnostics, Chlamydia MIF IgG assay instructions). All assays were performed blind to patient AMD *CFH*/*HTRA1* genotype status.

### Statistics

One hundred and ninety-nine patients with early and late AMD, and 100 controls without AMD were selected. Univariate analysis of differences between the AMD and control groups were performed using the χ^2^ test (and Fisher Exact test when appropriate) for categorical data. Continuous data was analyzed using the Student *t* test. Multivariate analysis was performed using binary logistic regression to assess whether covariates (including *CFH* Y402H/*HTRA1* genotype status, *Chlamydia* species serology status, age, sex, and BMI) could predict AMD status or severity. The level of statistical significance in the study was defined as p<0.05. When *Chlamydia* species were compared, a p value of <0.0167 was used to determine statistical significance, taking into account the Bonferroni correction for multiple testing. All statistical analyses were performed using SPSS (version 18.0, IBM, New York, NY).

## Results

### Baseline demographics and prevalence of *Chlamydia* infection

Baseline demographics are described in Table 1. Compared to controls, patients with AMD were older (78.3 versus 75.6 years, p=0.009), with a higher proportion of women (72.9% versus 56%, p=0.003). Overall, *C. pneumoniae* infection was common (69.2%), whereas *C. trachomatis* and *C. psittaci* were less prevalent (7.4% and 3.3%, respectively). There was no difference in age between seropositive and seronegative patients, for *C. pneumoniae* (78.1±8.1 versus 78.6±8.6, p=0.688) and *C. trachomatis* (80.1±6.3 versus 78.1±8.4, p=0.344), although patients seropositive for *C. psittaci* were older (84.0±8.0 versus 78.0±8.2, p=0.044). There was also no difference in age between patients with early versus late AMD (Table 2).

**Table 1:** Demographic data and *chlamydia* species seropositivity status in all patients and AMD versus controls

**Demographic data and *Chlamydia* IgG +ve status**	**Total**	**Control**	**AMD**	**p value (AMD versus non-AMD)**
Number	299	100	199	
Age (mean years±SD)	77.4 (±8.4)	75.6 (±8.4)	78.3 (±8.2)	0.009 #
Sex–males (%, n)	32.8% (98)	44.0% (44)	27.1% (54)	0.003 #
BMI (mean±SD, kg/m^2^; total n=295)	26.3 (±4.8)	25.7 (±5.0)	26.6 (±4.7)	0.148
*C. pneumoniae* IgG+ve (%, n)	69.2% (207)	70% (70)	68.8% (137)	0.838
*C. trachomatis* IgG+ve (%, n)	7.4% (22)	5.0% (5)	8.5% (17)	0.268
*C. psittaci* IgG+ve (%, n)	3.3% (10)	2.0% (2)	4.0% (8)	0.504*

AMD=Age-related macular degeneration, BMI=Body mass index, C.=*Chlamydia* #: Statistically significant NB: Student *t*-test (continuous variables)/χ^2 ^test (categorical variables) performed for all comparisons, except for those indicated by * (Fisher test used). P values for *Chlamydiae* species seropositivity associations are uncorrected.

**Table 2:** *Chlamydia* species seropositivity status in early versus late AMD

**Demographic data and *Chlamydia* IgG +ve status**	**Early AMD**	**Late AMD**	**p value (early versus late AMD)**
Number	53	146	
Age (mean years±SD)	77.4 (±9.8)	78.6 (±7.6)	0.372
*C. pneumoniae* IgG+ve (%, n)	58.5% (31)	72.6% (106)	0.057
*C. trachomatis* IgG+ve (%, n)	7.5% (4)	8.9% (13)	0.762 *
*C. psittaci* IgG+ve (%, n)	7.5% (4)	2.7% (4)	0.127 *

AMD=Age-related macular degeneration, C.=*Chlamydia* NB: Student *t*-test (continuous variables)/χ^2^ (categorical variables) performed for all comparisons, except for those indicated by * (Fisher test used). P values for *Chlamydiae* species seropositivity associations are uncorrected.

### *Chlamydia* and AMD

Univariate analysis demonstrated no statistical association between IgG seropositive status for all three *Chlamydia* species and AMD status. A subgroup analysis of the AMD group also demonstrated no association between *Chlamydia* species seropositivity and AMD severity (early or late; Table 2).

### *Chlamydia* and *CFH* genotype

No association was found between *Chlamydia* species seropositivity and *CFH* Y402H polymorphism, either when comparing genotypes (TT versus CT versus CC) or the presence or absence of the risk allele (C; Table 3). There was also no association between *Chlamydia* species seropositivity and AMD status or severity in those carrying the *CFH* Y402H risk allele (C; Table 4).

**Table 3:** Association of *Chlamydia* species seropositivity status and *CFH* genotype

***Chlamydia* IgG +ve status**	***CFH* Y402H status**	**% IgG +ve**	**p value**
*C. pneumoniae* IgG+ve (%,n)	TT	64.6% (66)	
	CT	70.0% (70)	0.434
	CC	73.0% (73)	
	C allele	71.5% (143)	0.227
*C. trachomatis* IgG +ve (%,n)	TT	8.1% (8)	
	CT	8.0% (8)	0.816
	CC	6.0% (6)	
	C allele	7.0% (14)	0.736
*C. psittaci* IgG +ve (%,n)	TT	2 (2.0%)	
	CT	4 (4.0%)	0.669*
	CC	4 (4.0%)	
	C allele	8 (4.0%)	0.370*

*CFH*=*Complement Factor H* gene, C.=*Chlamydia* NB: χ^2^ test performed for all comparisons, except for those indicated by * (Fisher test used). P values for *Chlamydiae* species seropositivity associations are uncorrected.

**Table 4:** *Chlamydia* species seropositivity status and AMD status/severity in *CFH* Y402H C allele carriers

	***CFH* Y402H risk C allele present**					
***Chlamydia* IgG +ve status**	**Control (n=67)**	**AMD (n=133)**	**p value (control versus AMD)**	**Early AMD (n=30)**	**Late AMD (n=103)**	**p value (early versus late AMD)**
*C. pneumoniae* +ve (%, n)	70.1% (47)	72.2% (96)	0.724	60.0% (18)	75.7% (78)	0.091
*C. trachomatis* +ve (%, n)	6.0% (4)	7.5% (10)	0.686*	6.7% (2)	7.8% (8)	1.000*
*C. psittaci* +ve (%, n)	1.5% (1)	5.3% (7)	0.272*	13.3% (4)	2.9% (3)	0.045*

AMD=Age-related macular degeneration, *CFH*=*Complement Factor H* gene, C.=*Chlamydia* NB: χ^2^ test performed for all comparisons, except for those indicated by * (Fisher test used). P values for *Chlamydiae* species seropositivity associations are uncorrected.

### *Chlamydia* and *HTRA1*

We also looked at patients carrying at least one *HTRA1* rs11200638 risk allele (A) to explore any association with *Chlamydia* seropositivity status and AMD. There was no association with *Chlamydia* species seropositivity and AMD status or severity in this group (Table 5).

**Table 5:** *Chlamydia* species seropositivity status and AMD status/severity in *HTRA1* rs11200638 A allele carriers

	***HTRA1* risk A allele present**					
***Chlamydia* IgG +ve status**	**Control (n=43)**	**AMD** **(n=122)**	**p value (control versus AMD)**	**Early AMD (n=31)**	**Late AMD (n=91)**	**p value (early versus late AMD)**
*C. pneumoniae* +ve (%, n)	72.1% (31)	69.7% (85)	0.765	67.8% (21)	70.3% (64)	0.787
*C. trachomatis* +ve (%, n)	7.0% (3)	11.5% (14)	0.563*	12.9% (4)	11.0% (10)	0.751*
*C. psittaci* +ve (%, n)	0% (0)	3.3% (4)	0.574*	9.7% (3)	1.1% (1)	0.050*

AMD=Age-related macular degeneration, C.=*Chlamydia* NB: χ^2^ test performed for all comparisons, except for those indicated by * (Fisher test used). P values for *Chlamydiae* species seropositivity associations are uncorrected.

### *Chlamydia*, *CFH* genotype, and AMD

Multivariate logistic regression analysis was then performed to identify covariates that could predict AMD status or severity. *C. trachomatis* and *C. psittaci* status was not included as covariates since IgG+ status was of low prevalence overall. When covariates were restricted to *C. pneumoniae* IgG status (positive versus negative) and *CFH* Y402H genotype (CC versus CT versus TT), *C. pneumoniae* IgG status was not predictive of AMD status (p=0.842) or AMD severity (p=0.078). The outcome was similar when the presence of the *CFH* Y402H risk allele (C: present versus absent) was used as a covariate instead of the *CFH* genotype.

Controlling for multiple variables (*C. pneumoniae* IgG status, age, gender, BMI, *CFH* Y402H genotype, *HTRA1* rs11200638 genotype), logistic regression analysis indicated predictors for AMD status included increased BMI (p=0.015, OR 1.08, 95% CI: 1.01–1.14), increased age (p=0.015, OR 1.04, 95% CI: 1.01–1.08), female gender (p=0.011, OR 2.04, 95% CI: 1.18–3.51), and *HTRA1* rs11200638 homozygous (AA) status (p=1.45×10^−4^, OR 11.05, 95% CI 3.20–38.17). Again, *C. pneumoniae* IgG+ve status (p=0.768) was not a predictor of AMD status.

We then performed logistic regression to predict whether these same covariates (*C. pneumoniae* IgG status, age, gender, BMI, *CFH* Y402H genotype, *HTRA1* rs11200638 genotype) were predictors of AMD severity. The only positive association in this model was *CFH* Y402H heterozygous (CT) status, associated with late AMD (p=0.006, OR 3.43, 95% CI 1.42–8.27).

## Discussion

In this study, there was no association with *Chlamydia* species seropositivity and AMD status or severity, either independently or when taking into account *CFH* and *HTRA1* risk genotypes, in addition to other variables (age, gender, BMI).

Only one previous study has reported a similar retrospective case-control study to ours, examining the association of *C. pneumoniae* status with *CFH* genotype on AMD status. Shen et al. looked at 148 AMD patients and 162 controls, and found an association between *C. pneumoniae* and AMD (OR 2.17, p<0.017), but not when a *CFH* risk genetic variant was taken into account. However, important differences exist between this study and ours. AMD patients were included if they had late AMD only (geographic atrophy/CNV), *C. pneumoniae* status was determined with PCR to identify *C. pneumonia* DNA in peripheral blood cells, and the rs380390 *CFH* single nucleotide polymorphism was selected as the risk *CFH* genotype. Furthermore, there was no comparison between AMD subtypes based on severity [42]. Haas et al. also performed a case-control study with 75 patients with AMD (any stage) and 75 controls, using enzyme-linked immunosorbent assay (ELISA) to determine *C. pneumoniae* seropositivity status. They found no association between *C. pneumoniae* seropositivity and AMD, or between *C. pneumonia* and the *CFH* Y402H genotype. This study did not perform further analysis of *C. pneumoniae* together with *CFH* genotype status in AMD due to insufficient numbers [41]. Baird et al. performed a prospective study following up 233 patients over a mean of seven years. The authors found patients were more likely to progress if serum *C. pneumoniae* IgG levels, as measured with ELISA, were at the upper tertile (42.5%) compared to the lower tertile (20.8%). They also found an additive risk of AMD progression in patients with the *CFH* Y402H risk allele (C) and upper tertile of *C. pneumoniae* titers (OR 11.8, p=0.005) compared to those with T allele and lowest *C. pneumoniae* titer [40].

Far less has been reported on the association of the other two *Chlamydia* species and AMD, which may be a reflection of the lower prevalence in the developed world. Kayalagu et al. performed a small case-control study looking at serum levels of *C. trachomatis* heat shock protein in 25 patients with AMD and 13 controls, and found no association [33]. Our study reached the same conclusions with a comparatively larger sample size, and additionally found no association with AMD risk genotype. No studies have investigated the association of *C. psittaci* and AMD. Although our study found no association between *C. psittaci* seropositivity and AMD, the low prevalence highlights the difficulty of studying this microorganism.

The prevalence of previous *Chlamydia* infection in this study group was higher for all three species compared to published data. This finding may reflect false positives from the MIF assay, or may indicate *Chlamydia* species prevalence rates are population-specific. PCR does have a higher specificity and sensitivity as a test for *Chlamydia* species, but was not chosen due to its reliance on the presence of *Chlamydia* DNA in the peripheral blood. Serological tests enable any previous infection to be detected, which was our aim. Serological techniques include complement fixation, indirect immunofluorescent assays, and ELISAs. The MIF test was chosen since it is considered the “gold standard” of *Chlamydia* serology testing [54]. Drawbacks of this method include the dependence on the binding antigen used, as well as variability in the experience and subjectivity of the person reading and interpreting the results [55]. The use of purified EBs to detect species and serovar-specific *Chlamydia* antibodies means cross-reactivity does not happen often with MIF but can occur especially between *C. psittaci* and *C. pneumonia* due to certain antigenic similarities [24,56].

In summary, *Chlamydia* infection status did not appear to be associated with AMD status or with severity in this study. The presence of *CFH* Y402H or *HTRA1* risk genotypes did not alter this negative association. The negative association reported here is consistent with some previous reports but ideally should be replicated in a larger AMD cohort.

## Appendix 1. Association of *C. pneumoniae* with AMD - summary of results from previously published studies.

To access the data, click or select the words “Appendix 1.” This will initiate the download of a compressed (pdf) archive that contains the file. Abbreviations: Early AMD: AMD excluding geographic atrophy (GA)/choroidal neovascularisation (CNV), Advanced/late AMD: Presence of GA/CNV, Wet AMD: Presence/past history of macular CNV, Dry AMD: Presence of macular drusen /pigmentary change/atrophy, OR: Odds Ratio, ELISA: Enzyme-Linked Immunosorbent Assay, IgG: Immunoglobulin G, IHC: Immuohistochemistry, N/A: Not Applicable, PCR: Polymerase Chain Reaction.
